# How a Long-Term Cover Crop Cultivation Impacts Soil Phosphorus Availability in a No-Tillage System?

**DOI:** 10.3390/plants13152057

**Published:** 2024-07-25

**Authors:** Hugo Mota Ferreira Leite, Juliano Carlos Calonego, Matheus Froés de Moraes, Lydia Helena da Silva de Oliveira Mota, Gustavo Ferreira da Silva, Carlos Antonio Costa do Nascimento

**Affiliations:** 1 Multidisciplinary Center, Federal University of Acre (UFAC), Forest Campus, Cruzeiro do Sul 69980-000, AC, Brazil; hugo.leite@ufac.br; 2 Department of Crop Science, School of Agriculture, São Paulo State University (UNESP), Botucatu 18610-034, SP, Brazil; juliano.calonego@unesp.br (J.C.C.); matheus.froes@unesp.br (M.F.d.M.); 3 Federal Institute of Education, Science and Technology of Acre (IFAC), Cruzeiro do Sul 69980-000, AC, Brazil; lydia.mota@ifac.edu.br; 4 Department of Soil Science, Luiz de Queiroz College of Agriculture (ESALQ), University of São Paulo (USP), Piracicaba 13418-260, SP, Brazil; cacnascimento@usp.br

**Keywords:** crop rotation, *Glycine max* (L.) Merril, no-tillage, occasional soil chiseling, phosphorus cycling

## Abstract

The growth of cover crops can contribute to the increase in phosphorus content at depth by root decomposition. The aim of this work was to verify the effect of cover crops on soil phosphorus availability and use by successive plants, and the accumulation of soil P in a no-tillage system conducted for 14 years. This research was carried out during the 2016/2017 and 2017/2018 crop seasons, whose treatments have been installed and maintained since 2003. The experimental design was a randomized block design, and the plots consisted of spring crops: pearl millet, forage sorghum, sunn hemp, and additionally, a fallow/chiseling area. The evaluation of available P was determined by P fractionation. In general, in the two years of evaluation, the accumulation of P in the shoot dry matter was higher in sunn hemp growth, on average 25% higher than pearl millet in 2016 and 40% higher than sorghum in 2017. The highest contents of labile inorganic P were in the sorghum–soybean and fallow/chiseling–soybean successions, with values higher than 50 mg kg^−1^ of P in the 0–0.1 m soil layer. However, in the other layers analyzed, the cover crops obtained higher availability of labile inorganic P. The systems using cover crops recovered 100% of the P fertilized in soybean.

## 1. Introduction

Soybean (*Glycine max* L. Merrill) is the main grain-producing crop in Brazil. However, most soybean-producing areas in Brazil are subject to P adsorption from soil colloids, thus reducing its availability to plants [1,2].

Brazilian soils under agricultural cultivation contain large reserves of P from the application of fertilizers, that is carried out with rates much higher than the crop needs, due to the P immobilization in the Fe- and Al-oxides and hydroxides present in highly weathered soils [3,4,5]. Research has been carried out to find solutions to access this reserve of P, which can meet the demand of the Brazilian crop fields for many years and, therefore, help to reduce the demand for P fertilizer [6,7,8].

The no-tillage system (NT), when using cover crops with grain-producing crops, promotes an increase in soil organic matter and P recycling, resulting in greater yield, either by accumulating residues on the surface or by root decomposition [2,9,10]. In no-tillage, due to the lack of turnover, most of the soil P remains accumulated in the top soil [11,12]; however, cover crops roots may improve P distribution throughout the soil profile, resulting in a better medium for the development of cash crops [13].

The accumulation of P in the crop residues determines the amount of P cycled by the crop, which can vary from 1 to 30 kg of P ha^−1^ (considering aerial and root biomass), depending on the species and the availability of P in the soil [14]. Cover crops such as pearl millet (*Pennisetum glaucum* L.), forage sorghum [*Sorghum bicolor* (L.) Moench] and sunn hemp (*Crotalaria juncea* L.) are grown throughout the country in different production systems [15,16], including in regenerative agriculture systems [17]. Janegitz et al. (2017), in research on the effect of cover crops on P availability, found that sorghum growth reduced P adsorption compared to pearl millet [18]. Studies have also shown that P recovery by sunn hemp for the subsequent crop was 1.6 times greater than millet [19].

In addition, cover crops can increase fertilizer use efficiency by reducing the fixation of P through the deposition of humic substances that block adsorption sites on the clay mineral surface [20]. The study of the vertical stratification of soil P forms, influenced by the growth and cultivation of cover crops in both the NT and direct seeding systems is important to understand the P dynamics in the soil. As the use of ground cover plants in the off-season makes it possible to improve soil fertility, studies on the interactions of long-term NT with cover crops on the dynamics and distribution of P in the soil profile are important to understand changes in the P lability caused by plants. Therefore, assessing soil P lability through phosphorus fractionation allows us to evaluate the effectiveness of a given cover crop species on the improvement of P availability to cash crops [6,21,22]. Thus, our aim in this work was to verify the effect of cover crop species on the availability and accumulation of P in the soil in a no-tillage system conducted for 14 years.

## 2. Results

### 2.1. Phosphorus Accumulation in Plants

**Figure 1:**
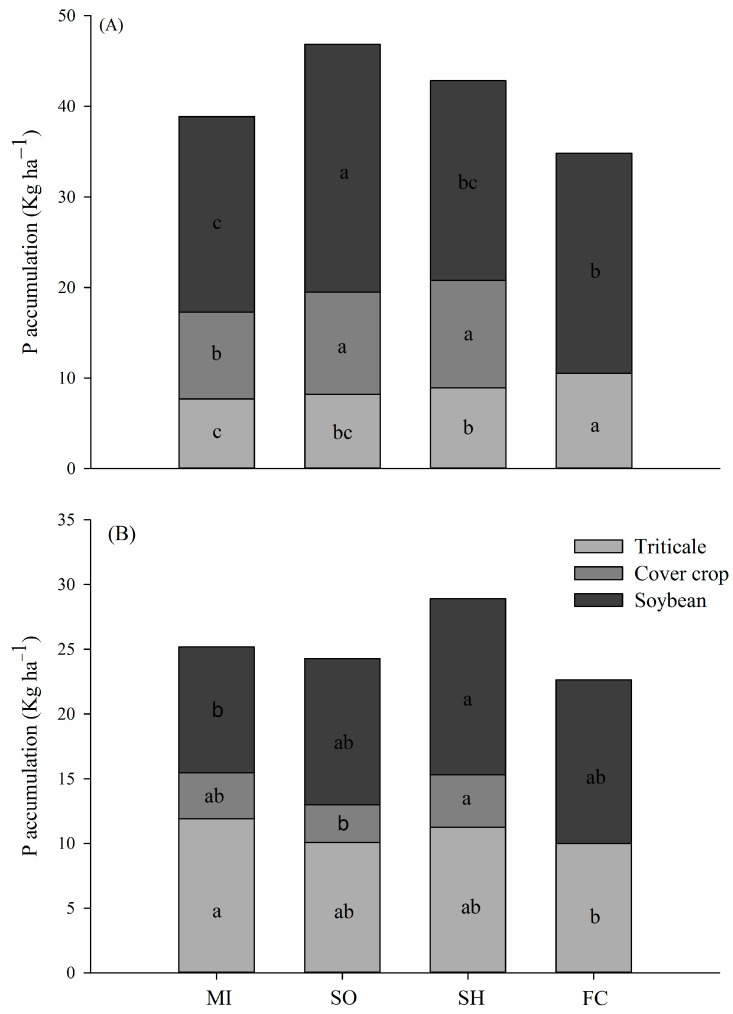
Phosphorus accumulation by soybean, triticale, and cover crops in first (**A**) and second (**B**) growing season. MI: millet; SO: sorghum; SH: sunn hemp; FC: fallow/chisel. Same letter denotes no significant difference between succession systems by LSD test at 5% significance level.

Phosphorus accumulation by triticale was higher in fallow/chiseling plots, being 36% higher than in millet (Figure 1A). Regarding P accumulation by the cover crops grown in spring, sorghum and sunn hemp reached higher amounts, accumulating about 2 kg P ha^−1^ more than millet. The accumulation of P by soybean in the first growing season was greatest in the succession sorghum–soybean, 27.3 kg ha^−1^, followed by soybean after fallow/chiseling, 24.3 kg ha^−1^ (Figure 1A).

**Figure 2:**
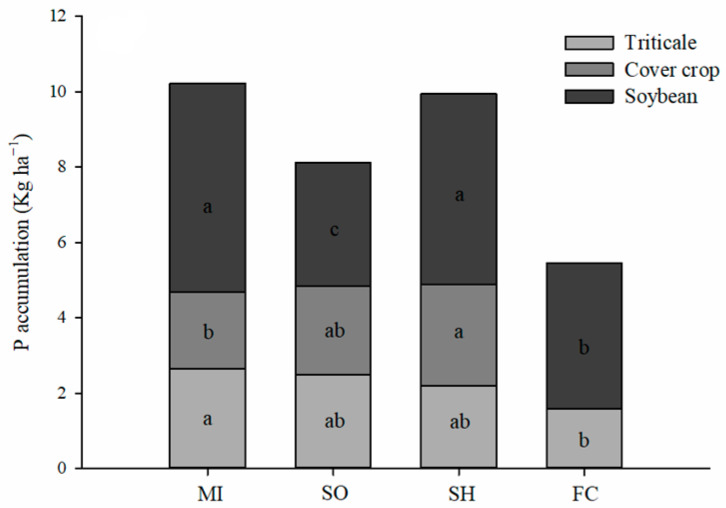
P accumulation in root dry matter in crop rotation systems during the 2016/2017 cycles. MI: millet; SO: sorghum; SH: sunn hemp; FC: fallow/chisel. Different lower-case letters for crop succession systems differ by the LSD test (*p* < 0.05).

Root P accumulation was higher in millet and sunn hemp plots, reaching on average 10 kg P ha^−1^, respectively (Figure 2). The P in the roots of each crop rotation system using cover crops in the spring showed an average accumulation of 73% in the 0.0–0.60 m layer in relation to fallow/chiseling (Figure 2). Results of P accumulation in soybean cultivation in the 16/17 crop season demonstrate, in general, high efficiency of crop succession systems in relation to P absorption by soybeans.

Crop rotation systems with cover crops showed greater efficiency in P accumulation, either through the accumulation of residues on the surface or the decomposition of roots in succession in agricultural crops, inferring that cover crops promoted the solubilization and/or less adsorption of P than soil (Figure 1 and Figure 2).

### 2.2. Phosphorus Lability in Soils

**Figure 3:**
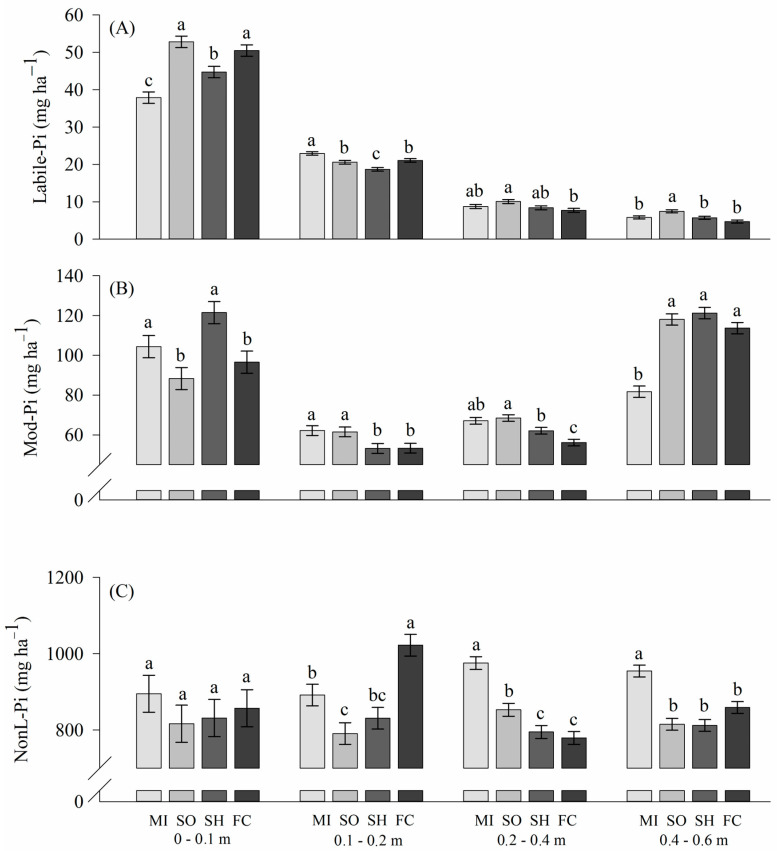
Labile inorganic P (**A**), moderately labile P (**B**) and non-labile P (**C**) in the soil profile as affected by millet (MI), sorghum (SO), sunn hemp (SH) and fallow/chisel (FC). Same letters for the crop succession systems within the same soil layer denotes no significant difference by the LSD test at 5% significance level.

The rotation systems affected soil P lability, showing differences between the cover crops grown in spring and fallow/chiseling (Figure 3). In the top soil (0–0.1 m depth), the highest levels of labile Pi (*p* < 0.05) were in the sorghum–soybean and fallow/chiseling–soybean sequences, with values greater than 50 mg kg^−1^ of P (Figure 3A).

In the 0.1–0.2 m soil layer, millet–soybean succession provided the highest labile Pi concentration. In the next layer, of 0.2–0.4 m, millet, sorghum and sunn hemp did not differ (*p* < 0.05), with concentrations between 8.4 and 10 mg kg^−1^ of labile Pi. In general, in depth, cover crops showed superior performance in the availability of labile Pi (*p* < 0.05) compared to fallow (Figure 3A).

In the moderately labile Pi (Mod-Pi) on the top layer, the effect of rotation systems was inverse to what happened in the labile fractions, specifically the millet and sunn hemp crops, which presented the lowest levels of labile Pi (Figure 3A); these crop successions promoted the conversion of labile Pi to Mod-Pi in the 0–0.1 m soil layer (Figure 3B). Also, the growth of millet and sorghum increased Mod-Pi by 17 and 21% in the layers 0.1–0.2 m and 0.2–0.4 m, respectively, in relation to fallow/chiseling (Figure 3B). Crops under cover crops obtained higher concentrations of Mod-Pi in the layers evaluated (*p* < 0.05), except in the 40–60 cm layer.

In the 0–0.1 m layer, the concentrations of non-labile Pi (NonL-Pi) did not differ significantly between treatments (Figure 3C). Millet improved NonL-Pi in subsurface layers (*p* < 0.05), i.e., with millet, the concentration of NonL-Pi was about 100 mg kg^−1^ higher than in the other treatments, regardless of soil layer, except for fallow/chiseling at 0.1–0.2, which provided the highest NonL-Pi content, i.e., 1000 mg kg^−1^ (Figure 3).

In general, the levels of labile Pi in the 0–10 cm layer correlate with Mod-Pi, that is, when there is an increase in the concentration of labile Pi, there is a reduction in Mod-Pi, and the opposite is also observed, depending on the management system (Figure 3A,B). For the other layers, the same behavior occurs between Mod-Pi and non-labile Pi (Figure 3B,C). In this way, a conversion of Mod-Pi to non-labile Pi is observed under millet cultivation from a depth of 20 cm. While, in the sorghum and sunn hemp growth, the opposite effect is observed, that is, a reduction in P adsorption occurs in the compartment with the lowest lability.

**Figure 4:**
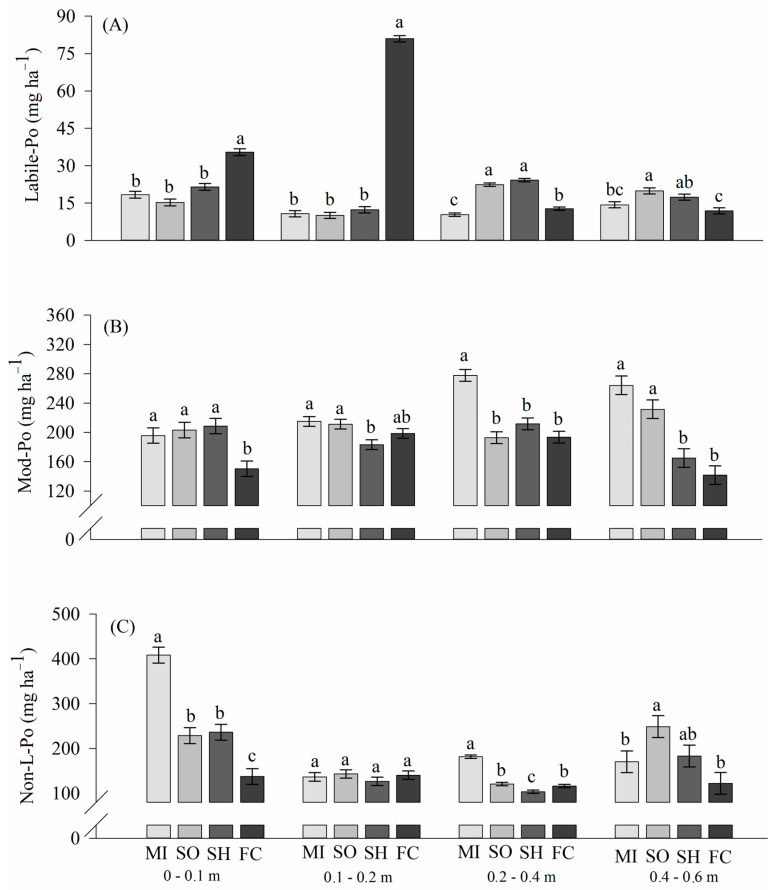
Organic soil P (labile (**A**), moderately labile (**B**) and non-labile (**C**)) as affected by millet (MI), sorghum (SO), sunn hemp (SH) and fallow/chisel (FC) in the layers of 0–0.1, 0.1–0.2, 0.2–0.4 and 0.4–0.6 m. Different lower-case letters for the crop succession systems at each depth differ by the LSD test (*p* < 0.05).

The tillage systems with cover crops in the spring differed (*p* < 0.05) from the fallow/chiseling system in the concentrations and forms of Po present in the soil in the evaluated layers (Figure 4). In the 0–0.1 m layer, in general, the highest lability of Po was in fallow/chiseling compared to cover crops. The growth of cover crops provided an increase in Po contents in moderately labile (Mod-Po) and non-labile (NonL-Po) pools, extracted by PoHid-0.1 and PoHid-0.5, respectively.

The concentrations of labile and Mod-Po among the cover crops in the 0–0.1m layer did not differ significantly (*p* < 0.05) (Figure 4).

The concentration of non-labile Po (NonL-Po) in the 0–0.1 m layer with the growth of millet was two-fold as compared to fallow/chiseling (Figure 4).

As in the previous layer, the same trend was noticed in the 0.1–0.2 m soil layer, with fallow/chiseling providing the highest content of labile Po, i.e., 81 mg kg^−1^ (Figure 4). The NonL-Po in fallow/chiseling did not differ from the succession systems with cover crops.

Beyond 0.2 m depth, sorghum and sunn hemp labile-Po content was on average 29.53, while it was 116.39 in fallow/chiseling (Figure 4). In contrast, millet provided the highest amount of both Mod-Po and NonL-Po beyond 0.2 m depth, but did not differ from sorghum.

The cover crops provided an increase in Po contents in the Mod-Po and NonL-Po compartments, extracted by Po_Hid-0.1_ and Po_Hid-0.5_, respectively.

In general, treatments with cover crops present a greater amount of Po than fallow crops, due to the sequential input through plant residues in spring crops; thus, the cycling P and/or increasing it in the storage compartments facilitates a lower lability.

**Figure 5:**
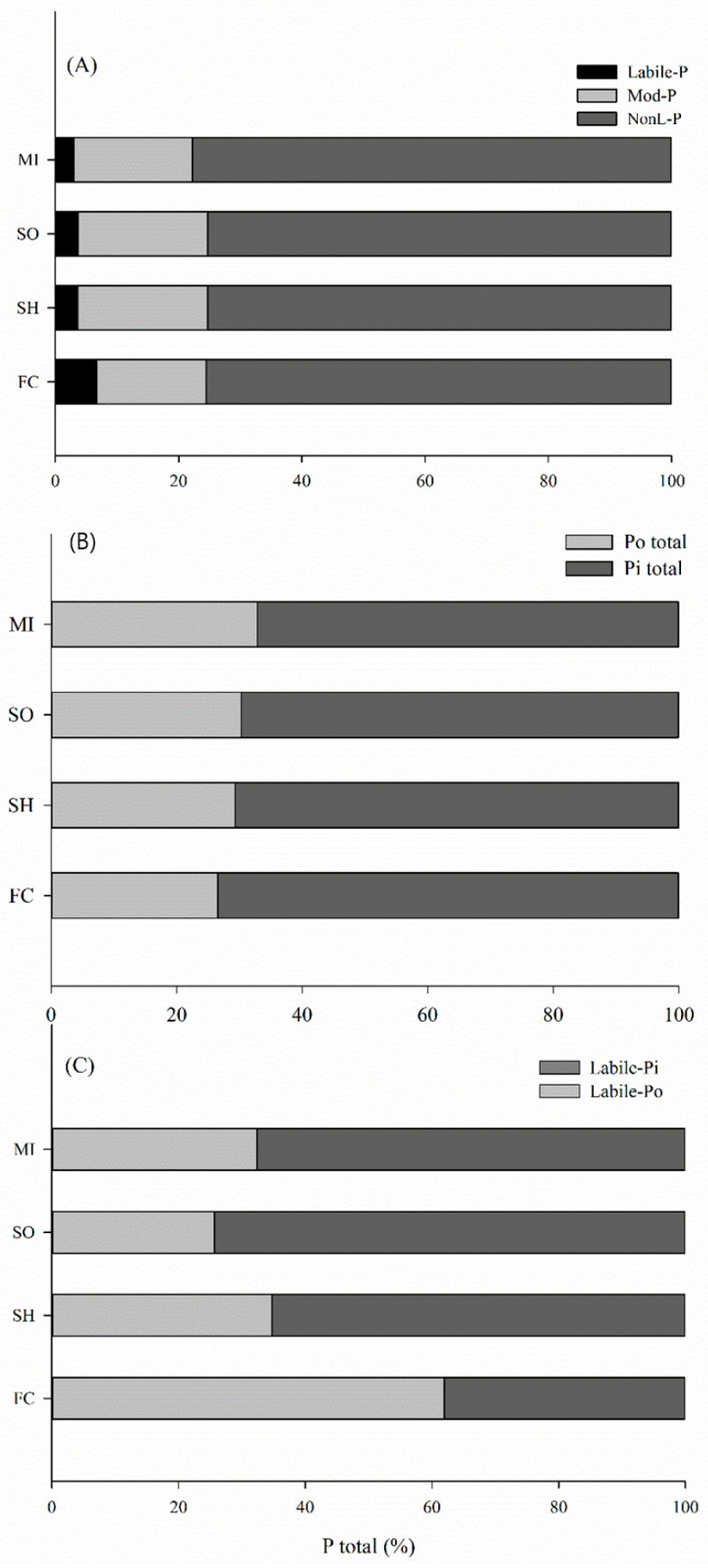
Relative contribution of labile, moderately labile and non-labile P (**A**), total organic P and total inorganic P (**B**) and labile organic and labile inorganic P (**C**) of the soil affected by crop rotations with millet (MI), sorghum (SO), sunn hemp (SH) and fallow/chisel (FC), in the layer of 0–0.2 m.

The effects of cover crops contrast in relation to the lability of Po from 0.2 m depth (Figure 5). While sorghum and sunn hemp promoted the greatest lability, millet increased the concentration of Po in forms of lower lability.

### 2.3. Distribution of Phosphorus into Soil Pools under Different Cover Crops

While with cover crops labile-P represented 3 to 3.6% of soil P, with fallow/chiseling it was 6.7% (Figure 5A). The relative contribution of Mod-P is around 17% in fallow/chiseling plots and on average 21% under sorghum and sunn hemp cover crops. The proportions of P forms in relation to the succession systems demonstrate that non-labile P was not affected by management, but labile P and Mod-P forms were influenced by the succession systems in the layer of 0–0.2 m (Figure 5A).

Among the cover crops, millet provided greater total Po accumulation in relation to fallow/chiseling plots, accumulating, respectively, 32 and 26% of the total P in the organic pool (Figure 5B). However, in all rotation systems, most of the total P was stored as inorganic P, thus, cover crops Pi represented 69% of the total P, while it was 74% with fallow/chisel. 

## 3. Discussion

### 3.1. P Accumulation by Plants

Sorghum and sunn hemp plants, grown in the spring season as cover crops, accumulated more phosphorus in their tissues (Figure 1A). The greater accumulation of P in the sorghum crop is due to the presence of the Sb03g006765 gene, which improves P uptake efficiency by increasing the root surface area [23]. In the case of sunn hemp, P uptake efficiency is improved by the release of organic acids that alters the lability P compounds around the rhizosphere, making them available for plant uptake [24].

In general, cover crops benefit the P nutrition of subsequent crops by different and simultaneous processes: the transfer of P in the decomposition of crop residues, and the exudation of organic acids and enzymes by the roots and microbial interactions providing P, via solubilization and mineralization [14], thus justifying the higher accumulations of P in soybean plants grown in these treatments, especially in crop rotations with sorghum.

The greater accumulation of P in the roots of cover crops is of total importance in the cycling of this nutrient in no-tillage. The roots of cover crops redistribute P in the soil profile, since in no-tillage there is no revolving of the soil, causing a new pore architecture of the roots and, with the decomposition of them, the release of organic and inorganic forms of P occurs [25,26,27]. In addition, most of the accounted P accumulated in the roots was found in soybean root, showing that cover crops not only help P redistribution in the soil profile, but also improve P nutrition for cash crops.

### 3.2. Phosphorus Lability: Inorganic Phosphorus

Even without P input, through crop residues in the spring growth, fallow/chiseling treatment in top soil (0–0.1 m depth) obtained labile Pi concentrations higher than the treatments of millet and sunn hemp (Figure 3A). Almeida et al. (2018), comparing nonfertilized soils, found the available P to be much lower in soil cultivated with Ruzigrass than in soil from fallow plots [6]. They attributed that to the formation of phytates under forage growth. In addition, chiseling improves organic P mineralization, raising the plant available P in soil; however, chiseling also has a negative impact, as it leads to the loss of soil organic matter [28].

However, only sorghum provided available Pi content significantly higher than fallow/chiseling beyond 0.20 m depth. Sorghum presents a root morphological alteration, expressed by the Alt SB gene, that confers tolerance to aluminum by the release of citrate [29]. Citrate is an organic acid that reduces P adsorption by occupying the adsorption sites on the surface of clay minerals [30,31].

In moderately labile Pi (Mod-Pi) on top layer, the effect of rotation systems was inverse to what happened in the labile fractions (Figure 3B). This denotes an effect of both sorghum and sunn hemp to improve the lability of P. This decrease in Mod-Pi becomes important due to its contribution to the labile P pool [32]. Pavinato et al. [33] reported organic acids exudated by the roots of plants such as millet and sorghum, and increased Mod-Pi levels in the 0–0.3 m soil layer. The growth of cover crops efficient in the absorption of P is important for the solubilization of the recalcitrant P, making it available for the subsequent culture [28].

The lower NonL-Pi values for the growth of sorghum and sunn hemp are due to the action of the roots that present a greater capacity in the solubilization of P, through the acidification of the rhizosphere, the exudation of organic acids and the secretion of extracellular phosphatase [34].

### 3.3. Phosphorus Lability: Organic Phosphorus

As growth under no-tillage does not incorporate crop residues, which remain on the surface with a small area of contact with the soil, decomposition is slow and thus reduces mineralization, being one of the factors responsible for the lack of changes in Po [35]. The concentration of Mod-Po in cover crops for fallow/chiseling, in the 0–0.1 m layer, reaches 45 mg kg^−1^ of Mod-Po. The accumulation of Po in more stable forms may reflect high microbial activity [36].

In the 0–0.1 m layer, crop rotation with millet reduced the labile Pi and increased NonL-Po in this layer (Figure 4). The higher amounts of NonL-Po in successions with cover crops can be explained by the sequential input of crop residues on the soil surface, resulting in the accumulation of recalcitrant Po [37]. Also, a high level of NonL-Po and Mod-Po after the growth of cover crops may be due to the deposition of inositol phosphate by crop residues and the complexation of this phosphate to clay minerals and organic humic acids [38,39].

In the treatment with fallow/chiseling, the highest levels of labile-Po in layers 0–0.1 and 0.1–0.2 m may have been due to the chiseling performed in 2016, up to 0.3 m deep (Figure 4). This management increases the macroporosity and O_2_ supply due to the chisel’s mechanical action on the soil structure, which increases the organic matter oxidation rate, thereby improving Po lability [40,41]. Santos and Tomm [42], assessing nutrient availability in different soil tillage systems, observed higher levels of P in the 0–0.1 m layer with the use of the chisel in relation to conventional tillage.

Overall, plots with cover crops have a higher Po amount than fallow/chiseling due to the sequential input of Po through plant residues in spring crops, performing P cycling and contributing to the increase in P levels in pools of lower lability. Foltran et al. [21] observed that the high deposition of plant residues contributes to the increase in moderately labile Po. The effects of cover crops contrast in relation to the Po lability from 0.20 m deep (Figure 4). While sorghum and sunn hemp promoted the highest lability, millet increased the Po content in the pools with less lability. The accumulation of less labile Po in soil depends on plant residue quality, that is, the higher the concentration of lignin and the C/N ratio, the higher the residue quality for covering soil [43].

### 3.4. Distribution of P into Pools at Arable Soil Layer

This sole assessment of the P pool contribution in the 0–0.2 m soil layer is very important, because it is the layer where we can find the highest percentage of cash crop roots, in addition to being the layer most affected by cultivation systems and also where most of P absorbed by plants comes from [44,45].

In this research, labile P values are below that found by Rodrigues et al. (2016), in no-tillage areas in the Brazilian savanna, i.e., for the same soil layer labile P represented approximately 20% of the total P [12]. These results demonstrate that the cover crops are keeping the P moderately labile, except for millet, which contributed more P in the non-labile fraction. Overall, regardless of the rotation system, most of the soil P was found as NonL-P, that is, about 75% total P were not readily available for plant uptake, at least not in the medium or short term (Figure 5A). Phosphorus is stored in the labile and moderately labile pools when the P inputs and outputs have a positive balance [46,47]. However, NonL-P pools may be used in succession systems with efficient cover crops, converting this reserve into a labile pool, being a key strategy to reduce P fertilizer demand and the profit of food production systems, mainly in soils with a high P adsorption capacity [5,28].

The highest total Po accumulation in the crop rotation with millet (Figure 5B) was driven by the NonL-P pool in the 0–0.1 m layer, where the P concentration was 408 mg kg^−1^ (Figure 4). However, in all rotation systems, most of the total P was stored as inorganic P; these results agree with those reported from many long-term studies [48], because the added Pi rapidly interacted with clay minerals and became sorbed or precipitated into Al and Fe [49], mainly in soils like those in our study (Table 1). In addition, the accumulation of organic matter in tropical soils is very slow, even in no-tillage systems [50].

## 4. Materials and Methods

### 4.1. Experimental Site

This research was carried out at an experimental farm located at Botucatu, SP, Brazil (22°48′57″ S, 48°25′41″ W; 786 m a.s.l.), during the 2016/17 and 2017/18 crop seasons. The soil is classified as Typic Rhodudalf [51]. The soil chemical and granulometric characteristics are shown in Table 1.

**Figure 6:**
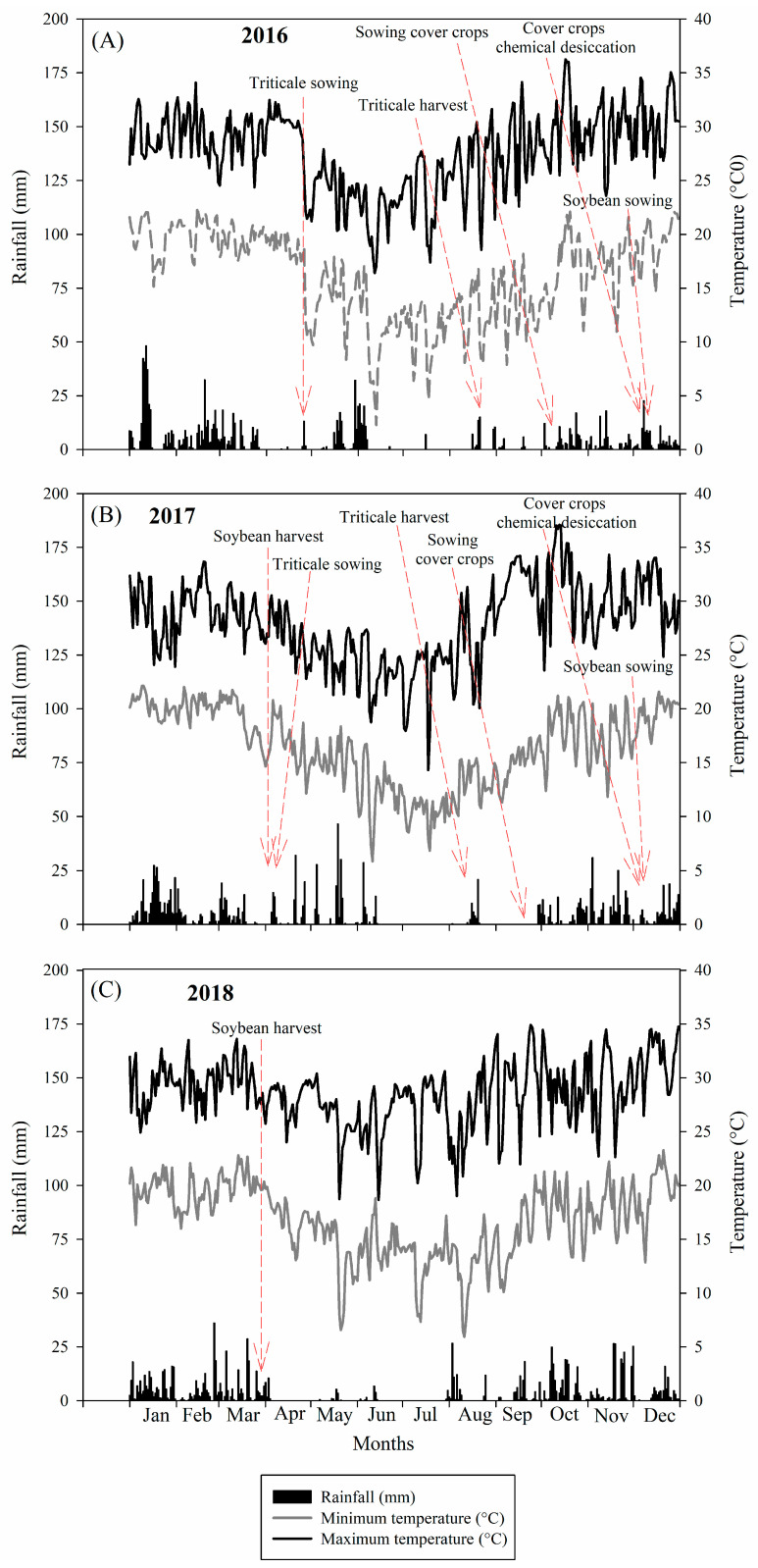
Rainfall and maximum and minimum temperatures recorded during the period from January to December 2016 (**A**), 2017 (**B**) and 2018 (**C**).

The climate of the region is mesothermal, with a dry winter (CWa); according to the Köppen classification, the dry season occurs between the months of May and September, and it has a mean annual rainfall of 1450 mm. The means for temperature and rainfall during the conducting of this experiment are shown in Figure 6.

### 4.2. Area History and Crop Rotations

The experiment has been carried out in NT since 2003, with crop rotations consisting of cover crops. In the fall/winter season, triticale (*X Triticosecale* Wittmack) was cultivated. After the triticale harvest and with the onset of spring rains (between September and October), the cover crops millet (*Pennisetum glaucum* L.), forage sorghum [*Sorghum bicolor* (L.) Moench] and sunn hemp (*Crotalaria juncea* L.) were cultivated, to produce straw for soybean cultivation. A fallow/chiseling area was maintained only in the spring season, without the presence of cover crops, and was chiseling with a depth of 30 cm in the years 2003, 2009, 2011, 2013 and 2016. In the summer, the soybean crop was cultivated.

Nitrogen fertilization was not performed on any of the crops involved in the crop rotation system. Fertilization with P and K was carried out in the sowing line only in soybeans. The phosphate fertilizations, carried out since the implementation of the experiment, were 30.5 kg ha^−1^ of P_2_O_5_ between the years 2003 and 2008; 26.2 kg ha^−1^ of P_2_O_5_ between 2009 and 2010; 22.1 kg ha^−1^ of P_2_O_5_ from 2011 to 2015; and 26.2 kg ha^−1^ of P_2_O_5_ between 2016 and 2017. The sum of phosphate fertilization over the years was approximately 400 kg ha^−1^ of P_2_O_5_, in the form of triple superphosphate. The potassium fertilization since the experiment was implemented totaled 50 kg ha^−1^ of K_2_O, in the form of KCl.

During the conducting of the experiment, liming was carried out in 2009 (1.8 t ha^−1^) and 2013 (2 t ha^−1^) on the experiment area.

### 4.3. Experimental Design and Treatments

The experiment was conducted in a randomized block design, with four replications. The treatments were spring crops (millet, sunn hemp, forage sorghum and fallow/chiseling). Each experimental plot was demarcated with a useful area of 40 m^2^ (5 × 8 m).

In the triticale crop (fall/winter crop), 29 sowing rows were installed with a spacing of 0.17 m between rows in each plot. In the cultivation of spring crops, 29 rows of millet, sorghum and sunn hemp were sown with a row spacing of 0.17 m. Soybean (summer crop) was sown in all plots with a row spacing of 0.45 m, resulting in 11 sowing rows. The distance between blocks and between plots was 4 m. The experiment used a total area of 736 m^2^ (46 × 16 m).

### 4.4. Experiment Conduction

The triticale crop (fall/winter crop) was sown during the two agricultural years on 25 April 2016 and 10 April 2017, without the use of fertilizers, using 170 kg ha^−1^ of seeds of the cultivar IPR 111. Formicide, based on Sulfluramid, was applied in the two sowings on 10 May 2016 and 30 April 2017. The control of broadleaf weeds was carried out with an application of 2.4 D (806 g a.i. ha^−1^) at the beginning of the tillering in both seasons. Triticale was harvested manually in both seasons on 16 September 2016 and 18 August 2017. The samples were mechanically threshed and the moisture content was adjusted to 13%, and the values were converted into kg ha^−1^. After the triticale harvest in both seasons, the chemical management of weeds was carried out through the application of the Glyphosate herbicide (720 g a.i. ha^−1^) on 23 September 2016 and 12 September 2017.

The sowing of spring plants was carried out during the two agricultural years on 5 October 2016 and 25 September 2017. For each sowing, 25 kg ha^−1^ of pearl millet seeds (cultivar BRS-1501), 30 kg ha^−1^ of sunn hemp seeds (cultivar IAC KR-1) and 20 kg ha^−1^ of sorghum seeds (hybrid Podium), were used. For the phytosanitary control of cover crops, Spinosad insecticide (14.4 g a.i. ha^−1^) was applied in both seasons on 10 November 2016 and 22 November 2017, and Sulfluramid-based formicide was applied according to need. The chemical desiccation of the cover crops occurred at pre-flowering on 10 December 2016 and 1 December 2017, through the application of Glyphosate (720 g a.i. ha^−1^).

The soybean cultivar TMG 7062 RR (summer crop) was sown in the 2016/2017 and 2017/2018 seasons on 15 December 2016 and 5 December 2017, respectively. Sowing was carried out with a population of 445 thousand seeds ha^−1^. Seeds were treated with the fungicide Carboxin + Thiran, the insecticide Tiametoxam, the inoculant *Bradyrhizobium* sp., and the micronutrients Co and Mo. Sowing fertilization was carried out with 50 kg ha^−1^ of K and 26.2 kg ha^−1^ of P_2_O_5_, in the form of potassium chloride and triple superphosphate, respectively. After soybean sowing in the 2017/2018 season, the experiment was affected by the dry season, resulting in lower soybean development, reflecting the lower yield compared to the 2016/2017 season.

Weed control in both seasons was carried out with the application of the herbicide Glyphosate (720 g a.i. ha^−1^) associated with the herbicide Sethoxidim (184 g a.i. ha^−1^) on 13 January 2017 and 10 January 2018. In the 2017/2018 season, the fungicide Pyraclostrobin + Epoxiconazole (0.08 + 0.03 kg a.i. ha^−1^, respectively) was applied preventively to control foliar diseases and the insecticide Thiamethoxam + Lambda-Cialotrin (0.028 + 0.21 kg a.i. ha^−1^) was applied to control stink bugs, on 16 January 2018. In both harvests, the fungicide Azoxystrobin + Cyproconazole (0.06 + 0.024 kg a.i. ha^−1^, respectively) and the insecticide Thiamethoxam + Lambda-Cialotrin (0.028 + 0.21 kg a.i. ha^−1^) were applied preventively to control stink bugs, on 24 January 2017 and 30 January 2018. Another preventive application of the fungicide Trifloxystrobin + Prothioconazole (75 + 87.5 g a.i. ha^−1^) and the insecticide Thiamethoxam + Lambda-Cialotrin (0.028 + 0.21 kg a.i. ha^−1^) was carried out to control stink bugs, on 23 February 2017 and 7 March 2017, and in the 2017/2018 season on 7 March 2018. Soybean was desiccated in both harvests with Paraquat (0.4 kg a.i. ha^−1^) on 3 April 2017 and 21 March 2018.

The soybean harvest in the 2016/2017 season was carried out on 6 April 2017 and in the 2017/2018 season on 28 March 2018. In both seasons, three central lines measuring 3 m in length were collected from each plot, making up an area of 2.7 m^2^, and the samples were mechanically threshed. The moisture content was adjusted to 13% and the values extrapolated to kg ha^−1^.

### 4.5. Plant Sampling and Analyses

Total P accumulation by triticale and soybean plants were sampled at harvest, while the cover crops were sampled at the pre-flowering stage before termination. Four subsamples per plot were collected using a 0.5 × 0.5 m rigid quadrat. The samples were oven dried at 65 °C for 72 h and weighed, ground and analyzed for P concentration [52]. The soybean and triticale grains were weighed separately for each shoot part, dried, grounded and had their P content determined [52]. The total P accumulation in the shoots for soybean and triticale was defined as the sum of the amount of P present in the shoot and grains; while for cover crops only the P accumulated in the shoot was determined, as they were terminated before flowering.

Root sampling for cover crops was carried out before termination in 2016 and for triticale and soybean it was conducted at the flowering stage in 2017. The roots were sampled using a 5 cm diameter steel probe. Four sub-samples were taken from 0 to 0.1, 0.1–0.2, 0.2–0.4, and 0.4–0.6 m soil. Then, the roots samples were washed, oven dried at 60 °C for 72 h, weighed, ground and analyzed for total P content [52]. Total P accumulated in the root was estimated by multiplying the P content in the roots by its dry mass weight, and the soil volume was sampled.

### 4.6. Soil Collections and Analyzes

Before soybean sowing in the 2017/2018 season, in November, soil was collected at depths of 0–10, 10–20, 20–40 and 40–60 cm. The samples with deformed soil structure were composed of four subsamples per plot, which were collected using an auger hole, and placed in plastic bags. The samples were sieved through a 2 mm mesh, air-dried and stored at room temperature, and then the soil phosphorus fractionation analyzes were carried out.

#### Soil Phosphorus Fractionation

Sequential P fractionation followed the methodology proposed by [53] with modifications by [54]. Briefly, 0.5 g of soil was added to centrifuge tubes. Next, sequential extraction was carried out as follows: anion exchange resin (P_AER_), then 0.5 mol L^−1^ NaHCO_3_ (P_Bic_), then 0.1 mol L^−1^ NaOH (P_Hid0.1_), then 1 mol L^−1^ HCl (P_HCl_) and then 0.5 mol L^−1^ NaOH (P_Hid0.5_). The residual P was determined by taking 0.1 g of the soil that remained after the sequential extraction, subjecting it to sulfuric digestion [55]. The alkaline extracts, i.e., P_Bic_, P_Hid0.1_ and P_Hid0.5_ were divided into two aliquots. In one of them, inorganic P (Pi) was colorimetrically determined according to the method of Dick and Tabatabai [56], modified by [57]. In the other aliquot, the total P was determined by sodium persulfate and a sulfuric acid digesting block [58], with modifications proposed by [59] and with adaptations by [60]. The phosphorus in acid extracts (P_AER_, P_HCl_ and P_residual_) and the total digestions of PBic, _PHid0.1_ and P_Hid0.5_ were colorimetrically determined [61]. The organic P (Po) in the alkaline extracts was calculated by the difference between total P and Pi.

The fractions of P were grouped into labile Pi (Pi_AER_ + Pi_Bic_); labile Po (Po_Bic_); moderately labile Pi (Pi_Hid0.1_ + Pi_HCl_); moderately labile Po (Po_Hid0.1_); non-labile Pi (Pi_Hid0.5_ + P_residual_) and non-labile Po (Po_Hid0.5_). In addition, the soil P was separated into biological and geochemical origin using the mean values of the fractions of the 0–0.2 m soil layer. The biological fractions consisted of the sum of the organic fractions of P and the geochemical fraction was grouped into all the inorganic fractions and P_residual_ [12,62].

### 4.7. Data Analysis

All data were normally distributed (W > 0.90) according the Shapiro–Wilk Test using UNIVARIATE procedure (version 9.3; SAS Inst. Inc., Cary, NC, USA). Cover crops were considered as a fixed effect. All dependent variables were subject to One-way ANOVA (cover crop effect). Fisher’s least significant difference (LSD) test was used to compare means. Statistical significance is reported at the 5% level of significance.

## 5. Conclusions

The accumulation of P by the roots of the succession systems demonstrates the biological incorporation of P at depth by the intense cultivation of plants.

The availability of P in the soil after 14 years of the succession of crops under the no-tillage system was modified by the growth of cover crops. The millet reduced the labile Pi in soil surface and increased the moderately labile and non-labile Po along the soil profile.

In general, the crop succession systems with cover crops promoted an increase in the labile P compartment in the inorganic fraction in the 0–0.2 m layer. However, in the succession system with fallow/chiseling, the labile P compartment was composed mainly of the organic fraction.

## Figures and Tables

**Table 1:** Chemical characterization in 2003 and granulometric distribution in 2017 of the soil in the experimental area.

Depth	pH	SOM	P_resin_	H + Al	K	Ca	Mg	Sand	Silt	Clay
m	CaCl_2_	g dm^−3^	mg dm^−3^	mmol_c_ dm^−3^				g kg^−1^		
0.0–0.1	5.0	29.5	31.5	73.5	3.8	32.9	13.5	107	238	655
0.1–0.2	4.6	25.9	15.2	97.2	2.5	35.2	15.7	100	245	655
0.2–0.4	4.8	22.4	3.3	68.7	1.1	46.3	15.0	84	211	705
0.4–06	5.1	22.0	2.3	63.5	0.2	56.7	11.5	66	204	730

pH: active acidity; CaCl_2_: 0.01 M calcium chloride solution; SOM: soil organic matter; P_resin_: exchangeable phosphorus; H + Al: potential acidity; Ca: exchangeable calcium; Mg: exchangeable magnesium; K: exchangeable potassium.

## Data Availability

Data is contained within the article.
